# Synergistic Synthetic Biology: Units in Concert

**DOI:** 10.3389/fbioe.2013.00011

**Published:** 2013-10-16

**Authors:** Jean-Yves Trosset, Pablo Carbonell

**Affiliations:** ^1^Sup’Biotech, BIRL, Villejuif, France; ^2^BioRetroSynth Laboratory, Institute of Systems and Synthetic Biology, University of Evry-Val d’Essonne, Evry, France; ^3^BioRetroSynth Laboratory, Institute of Systems and Synthetic Biology, CNRS, Evry, France

**Keywords:** synthetic biology, synergy, polypharmacology, multi-drug resistance, metabolic engineering, metabolic networks, synthetic lethality, coupling

## Abstract

Synthetic biology aims at translating the methods and strategies from engineering into biology in order to streamline the design and construction of biological devices through standardized parts. Modular synthetic biology devices are designed by means of an adequate elimination of cross-talk that makes circuits orthogonal and specific. To that end, synthetic constructs need to be adequately optimized through *in silico* modeling by choosing the right complement of genetic parts and by experimental tuning through directed evolution and craftsmanship. In this review, we consider an additional and complementary tool available to the synthetic biologist for innovative design and successful construction of desired circuit functionalities: biological synergies. Synergy is a prevalent emergent property in biological systems that arises from the concerted action of multiple factors producing an amplification or cancelation effect compared with individual actions alone. Synergies appear in domains as diverse as those involved in chemical and protein activity, polypharmacology, and metabolic pathway complementarity. In conventional synthetic biology designs, synergistic cross-talk between parts and modules is generally attenuated in order to verify their orthogonality. Synergistic interactions, however, can induce emergent behavior that might prove useful for synthetic biology applications, like in functional circuit design, multi-drug treatment, or in sensing and delivery devices. Synergistic design principles are therefore complementary to those coming from orthogonal design and may provide added value to synthetic biology applications. The appropriate modeling, characterization, and design of synergies between biological parts and units will allow the discovery of yet unforeseeable, novel synthetic biology applications.

## Introduction

Synthetic biology is an emerging discipline that proposes the application of engineering principles to design and construct biological systems with innovative and useful functionalities, usually not found in nature. To that end, synthetic biology circuits and devices are basic functional units obtained through the composition of genetic parts that are used in order to build more complex biological systems. Many successful synthetic biology applications have been reported to date, including genetic circuits with specific transfer functions such as oscillators, delayers, inducers, and synthetic metabolic pathways (Wang and Buck, 2012). Due to its engineering roots, the concept of modularity is central to the synthetic biology practice, as modularity is regarded as a key feature that can make biology close to synthetic disciplines such as computer science and engineering (Hartwell et al., 1999; Alon, 2003). Modularity dictates that circuits or devices should be designed in a way that allows their operation to be performed in a modular or plug-and-play fashion, thus allowing the reuse of these modules in different and often unrelated applications. To that end, synthetic biology modules try to satisfy as much as possible the condition of orthogonality, in which modules work as functional units whose intrinsic properties are independent of their environment (Lucks et al., 2008). Such goal is attained through the adequate attenuation of cross-talk between the elements of the circuits and the host or chassis organism (Del Vecchio et al., 2008). Modules built in that way should meet a standard collection of specifications allowing the application of automated design techniques to synthetic biology devices (Kitney and Freemont, 2012). One of the striking features of synthetic biology applications is their capacity for innovation by implementing new functionalities that were not observed before, at least in their final form, in natural biological systems (Endy, 2005; Lanza et al., 2012). Such innovative behavior is obtained through the rewiring of genetic elements in rather creative ways that often lead to unexpected emergent properties (Benner and Sismour, 2005). Notably, the resulting new behavior can in many cases be regarded as the by-product of a powerful latent property that is present among biological systems, namely the faculty of inducing synergies between constitutive elements toward the emergence of novel or reinforced activities. Even when direct relationships between units are minimized, indirect dependences are still possible to exist in which synergies may arise. Synergy, thus, is a concept inherent to modular systems, which has however not yet been exploited in synthetic biology to the same level of maturity as in other allied fields. By learning how synergies are unveiled and used, not only in nature but also in many technologically related disciplines such as drug design or metabolic engineering, we might pave the road toward accelerated innovation in synthetic biology applications. Various examples of synergies exist in nature and, in biology, synergy has been recognized as a driving force in evolution (Corning, 2013). Examples of synergies in biology include cooperative interactions between genes or the concerted effect of individuals in bacterial colonies (Wintermute and Silver, 2010). Synergies of scale, functional complementarities, symbiotic relationships, division of labor, etc., are also found everywhere in living systems. Synergies can provide an evolutionary advantage, like in secondary metabolites in plants that act synergistically against pathogens (Challis and Hopwood, 2003), or in the case of the emergence of resistance during multi-drug therapy (Hegreness et al., 2008). Certain genes act in synergy in the expression of a given phenotype. For a set of alleles, synergistic epistatic interaction is defined when the concerted effect of mutants is stronger than the “sum” of the effect of each mutant if considered as independent (Nordwald et al., 2013). Similarly, antagonist epistasis (equivalently alleviating or buffering synergy) is defined when the effect of all mutants together is less severe than the sum of the effect of single mutations (Perez-Pinera et al., 2013). This additive model for null-hypothesis independency is often used when the effect of each mutation can be measured with a log-type scoring such as in measuring drug efficacy for example. Otherwise, multiplicative or min models are used (Mani et al., 2008). In pharmacology, a synergistic/antagonistic drug combination is defined when the efficacy of two drugs is larger/smaller than the sum of the individual effects. The Loewe (additive) and Bliss (multiplicative) models are the corresponding independency models for drug combinations (Cokol et al., 2011).

This review explores how synergies are exploited in several disciplines for a possible adoption of such an approach in synthetic biology applications. The benefits will be multiple, since taking advantage of the synergies found in biological systems will allow us to develop innovative, efficient, and more reliable synthetic biology devices. In the next sections, we will first define first the concept of synergy, giving a formal definition, and then we will look at several techniques used for predicting synergies *in silico*. We will then review important cases of synergies found in drug design, toxicity, and metabolic engineering, as well as the main instances in synthetic biology where synergies were involved as major components in the conception of innovative applications.

## The Roots of Synergy in Biology

Synergy is a general mechanism that reflects the concerted action of two or several factors on a given outcome of a system. This concerted action produces an amplified or a cancelation (negative amplification) effect compared to the effect produced by each factor individually. Synergy can be defined in multiple ways depending upon the underlying model. In general terms, a property *x* has synergistic effects on an observed activity *f*  (*x*) in the system if the dependency of the property with respect to the activity shows superadditivity:

$$f\left(x_{1}+x_{2}\right)\geq f\left(x_{1}\right)+f\left(x_{2}\right)$$

Synergy can be in that way measured as the difference between the overall effect and the sum of the individual effects:

$$\text{Syn}\left(x_{1},x_{2};f\right)=f\left(x_{1}+x_{2}\right)-\left(\;f\left(x_{1}\right)+f\left(x_{2}\right)\right)$$

More interestingly, synergies can also be defined in the context of aggregated effects from a set of elements. Two subsets *X*_1_ and *X*_2_ from a set *X* display synergistic effects for the activity *f*  (*A*) when this set function *f* shows supermodularity (Topkis, 1998):

$$f\left(X_{1}\cup X_{2}\right)+f\left(X_{1}\cap X_{2}\right)\geq f\left(X_{1}\right)+f\left(X_{2}\right)$$

$$\text{Syn}\left(X_{1},X_{2};f\right)\text{ = }f\left(X_{1}\cup X_{2}\right)+f\left(X_{1}\cap X_{2}\right)-\left(f\left(X_{1}\right)+f\left(X_{2}\right)\right)$$

For instance, the superadditivity and supermodularity models correspond respectively to the Loewe additivity model and Bliss multiplicative models of independent drug effect in drug synergy (Cokol et al., 2011). In an information theory framework, synergy between two factors *X*_1_ and *X*_2_ with respect to the activity *f* is defined by the difference between the mutual information of each factor simultaneously and independently with respect to *f* (Anastassiou, 2007):

$$\text{Syn}\left(X_{1},X_{2};f\right)\text{ = }I\left(X_{1},X_{2};f\right)-\left(I\left(X_{1};f\right)+I\left(X_{2};f\right)\right)$$

**Figure 1:**
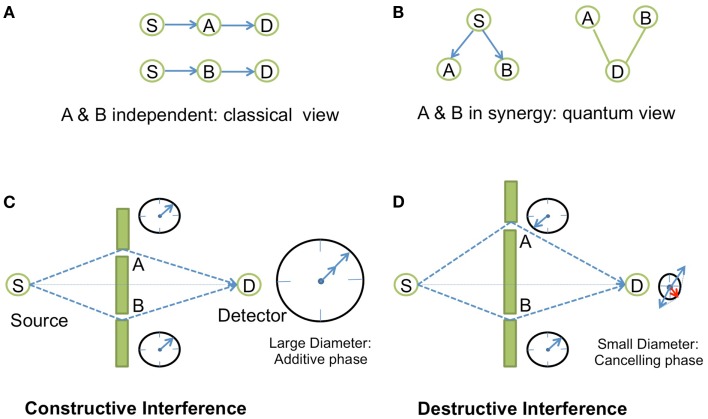
**Synergistic effects in Young’s interference experiment**. In Young’s experiment, a source *S* sends photons to a detector *D* that is located behind a wall with two tiny slots *A* and *B* on each side of the line between *S* and *D*. **(A)** When the two slots *A* and *B* are independent (by disconnecting the entanglement in measuring the photons emerging from one slot with a detector behind *A* or *B*), there is no interference, and the distribution of photons on the *z*-direction of the screen is just Gaussian. This is the classical view of the experiment. **(B)** In quantum view, using amplitude of probability, the photon emerging from *S* and going to *D* has two alternating ways: slot *A* or slot *B*. The probability of the total event is the square of the amplitude of probability given by the sum of the two amplitudes for each alternating event *Prob*(*S*–*D*) = |⟨*S*–*A*–*D*⟩ + ⟨*S*−*B*−*D*⟩|^2^, where ⟨.⟩ is the amplitude of probability of the event. **(C)** The probability of the total event will correspond to an amplified synergistic effect if the two arrows of the two independent events points toward the same direction, i.e., if the photons are in phase between each other which occurs when the difference between the two trajectories corresponds exactly to an integer number of time period difference between the two photons. This is symbolically represented by a “watch clock” for each photon that turns with a specific speed depending upon the frequency of the light. If the arrows of these two watches point toward the same direction, then the photons are in phase, and we get constructive interferences. **(D)** Destructive interference is observed when the arrows are in the opposite direction (opposite phase).

In physics, specially in Quantum Electrodynamics (QED) (Feynman, 1988), synergy is described in terms of phase concordance between two events, for instance in the case of light interference, through the concept of amplitudes of probability represented by complex numbers (Box 1; Figure 1). Similarly, the phenomena of resonance as seen in spectroscopy or in phase transitions captures the notion of synergy. This coupling phenomenon of resonance is also used in electronics in order to build oscillator circuits. The basic principle consists of establishing a synergy between two filtering elements through a feedback loop that positively amplifies the signal at the frequency of resonance. An oscillator circuit is obtained when the feedback effect cancels the damping effect, so that any small noise perturbation is enough to make the system start to oscillate. Oscillators following that principle are present in many biological systems. Natural oscillators include circadian rhythms, heartbeats, as well as gene expression, metabolic, and neuronal systems (Hess, 2000). Similarly to the electronic circuit oscillator, they are internally controlled through regulatory feedback loops (Bell-Pedersen et al., 2005; Ihekwaba et al., 2005), a concept that has been exploited in synthetic gene oscillators by using activator/repressor systems (Atkinson et al., 2003; Stricker et al., 2008; Tigges et al., 2009).

Box 1 **Synergy in Young’s experiments.**

In the QED theory, to any given event corresponds an amplitude of probability, a complex number |*r*|*e^i^*^θ^, the square of which gives the probability of this event to occur. On a complex plane, this complex number is represented by a vector of length |*r*| and angle θ with respect to the real axis. Two vectors pointing toward the same direction have the same phase. Synergy comes into play when we decompose the event into sub-events. When we add or multiply the amplitudes of probabilities of each sub-event (depending on whether the sub-events happen in alternative or in independent successive ways), the resulting vector may have a greater module if all the vectors of the sub-events point more or less toward the same direction, i.e., when all the vectors have similar phases. The canceling effect arises for example, when an event is decomposed into two alternating (parallel) and equivalent sub-events that are in opposite phases. Such synergistic positive or negative amplification effects could be observed in the famous experiment of light interference by Young (Figure 1). The message of this example is that all physical phenomena concerning photons and electrons are explained in QED in terms of amplitude of probability. The complex number view of probability seems to be an ideal tool at least in QED to study synergy.

Other examples are the divergence or amplified effect of thermodynamical variables observed in critical phenomena. At phase transitions, a “resonance” can be seen between the macroscopic and microscopic variables at the different length scales present in the system. Nobel laureate physicist by K. Wilson (Kadanoff, 2013) found a way to connect them through a hierarchical framework using a zooming multiscaled parameter (Yeomans, 1992). This concept of scaling has a nice correspondence in biology and is often named as an emergent property. For example, some properties of the lichen emerge when bacteria and microalgae enter in synergy (symbiosis). Conceptually, this means that at certain conditions of the system the properties of the “forest” do not emerge from studying the properties of the “tree” alone. In biology this scaling property suggests a hierarchical embedding from a single cell to a full organism. Scaling is therefore an additional concept to be integrated in synthetic biology to study, at certain critical conditions, for instance the emergent properties of a population of cells from a single cell.

## *In silico* Prediction of Synergies in Biological Networks

It is increasingly clear that complex interconnections between components can lead to the emergence of global effects resulting from a synergy between components or from a concerted ensemble of components called modules. This modular view of biological systems introduces the notion of synergy between modules at a given hierarchical level but also between different hierarchical levels. In analogy with phase transition at critical state, where all length scales are present (Yeomans, 1992), biological systems might reveal synergies between various modular hierarchical levels that explain global biological functions of the entire system (or dysfunctions like in the case of disease states in the organism).

We can think of two types of synergies: the “horizontal” one that corresponds to the concerted actions of modules at a given hierarchical level; and the “vertical” one that can be viewed as a resonance effect between components or modules at various hierarchical levels. Depiction of synergy depends therefore on the way the biological system is represented. Graph modeling of biological networks captures the relationships between the components, usually at a given length scale. These relationships are usually modeled from the local interactions observed experimentally such as protein–protein interactions or metabolic networks. The goal of the *in silico* model is to unravel the indirect effects or dependences within this large set of interacting components. *In silico* prediction of synergy is therefore based on two components: (i) hierarchical and/or modular description of the biological network; (ii) implicit or explicit incorporation of the biological response into the model.

Biological response can be included implicitly into the model by constructing for example a disease genes network when looking for a synergy between drug actions (Vitali et al., 2013), or explicitly as for example with a cost function in flux optimization of metabolic networks or as a statistical probability of a functional node in a Bayesian Network approach. Once a biological response is incorporated into the biological network, synergy between components can be deduced directly from the graph topology property of the biological network or by inferring the behavior of conjugate agents on the output response of the system from metabolic fluxes (see Metabolic Synergies below) or Bayesian Network approaches.

The graph-topological approach to explain synergy consists of establishing a relation between the graph property and the biological response in order to define a synergy score from topological graph descriptors. In the case of polypharmacology, the response is modeled through a bi-partite graph and corresponds to the effect of a molecule (drug) on a target or on a disease. This bi-partite graph is made of two kinds of nodes (e.g., drug nodes and target nodes) with no link between nodes of the same class and can therefore be seen as an interacting graph between the two class of elements, drug and target in this case. In order to deduce the neighborhood description amongst nodes of a same class, like the similarity of behavior of drugs or targets, a co-graph from the bi-partite graph is constructed that connects two nodes of the same class if they share the same partner in the other class. For example, two drugs are connected in the drug-graph if they interact with the same target. Similarly, two targets are connected in the target-graph if they bind a given drug (Yildirim et al., 2007; Li et al., 2011). Li et al. (2011) deduces synergistic drug combinations by analyzing the corresponding disease gene network for that specific disease. Their method, called NIMS (Network target-based Identification of Multicomponent Synergy), calculates drug synergy score using graph-topological descriptors that reflect the type of connection a drug-related gene (node) is making with the rest of the gene network. It includes graph-topological notions like hubs and betweenness centrality (Li et al., 2011; Vitali et al., 2013). This approach has been tested on a set of five agent pairs with known synergy in every 62 pairs for a given agent in the therapeutic area of angiogenesis and rheumatoid arthritis.

**Figure 2:**
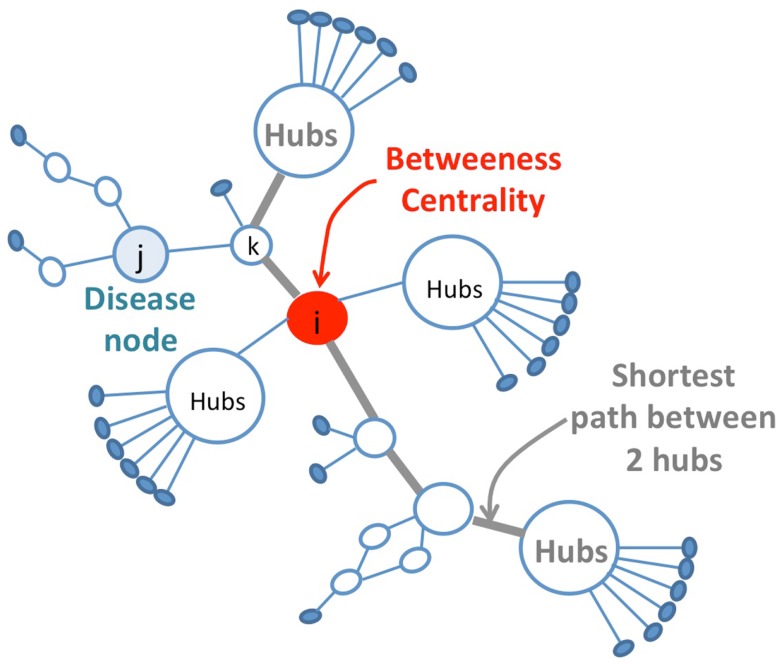
**Selection of protein candidates for pharmaceutical synergy**. The selection of source proteins that are potential candidates for pharmaceutical synergy is based on topological properties, favoring the in-between nodes with respect to hubs. Hubs proteins are highly connected nodes that tend to be more essential than non-hub proteins but targeting them might produce a large number of side effects. The drug combination strategy consists of targeting less important proteins to have less impact on the overall system alone while maintaining drug efficacy thanks to the synergistic effect on several points of the biological network. These secondary proteins correspond to in-between nodes in the network. They are identified using bridging centrality index that is computed as the product of its betweenness centrality and a bridging coefficient that evaluates if a node is located between high degree nodes (Table 1).

Another topological approach presented by Vitali et al. (2013) proposes an efficient identification of multicomponent synergies that rely on the particular topological properties of the nodes (Disease-Protein) in the disease network that are constructed from protein–protein interactions and expression data for the diseases under study. The selection of potential protein candidates for pharmaceutical synergy is based on topological properties of the network (Table 1; Figure 2). Then, a set function called Topological Score of Drug Synergy (TSDS) assigns a score to each combination of secondary proteins. Such a score is computed for each triplet of secondary proteins as the product of the node reachability index computed between the protein and each disease node (Table 1). These biological network-based approaches provide proof of concepts that will be further validated as larger sets of drug pairs with known experimental synergies become available for set of diseases.

**Table 1:** **Synergy score estimation from network topological properties**.

Type of score	Definition	Example of application
Bridging centrality	*BR*(*p*) = *B*(*p*) × *BC*(*p*)	Network-based characterization of drug-related genes (Kotlyar et al., 2012)
	*B*(*p*) is the betweenness centrality given by the fraction of shortest paths between node pairs that pass through the node *p*	
Bridging coefficient	$BC\left(p\right)=\frac{d{\left(p\right)}^{-1}}{\sum_{v\in N\left(p\right)}d{\left(v\right)}^{-1}}$	Network identification of robust bridged nodes (Hwang et al., 2008)
	Discriminates if a node *p* connects hub nodes, based on the degree *d*(*p*) of neighbors *N*(*p*)	
Node reachability index	$DP(p)=\sum_{d=1}^{N_{d}}\frac{\sum_{i=1}^{N_{sh}(p,d)}\Pi_{i,j}Wij}{N_{sh}(p)}$	Network-based prioritization of drug targets (Chua et al., 2011)
	Sum of the weights *w_ij_* of each shortest pathway between the source protein *p* and the diseases *d* divided by the number of pathways *N_sh_*	
Topological score of drug synergy	$\mathrm{TSDS}\left(p_{1},p_{2},p_{3}\right)=\prod_{p\in \left(p_{1},p_{2},p_{3}\right)}\mathrm{DP}\left(p\right)$	Network-based target ranking for polypharmacological therapies (Vitali et al., 2013)
	Product of the reachability index *DP*(*p*) for each triplet of source proteins (*p*_1_, *p*_2_, *p*_3_)	

## Synthetic Lethality: Synergies as Targets

A class of synergy observed in biological systems that has received much attention is synthetic lethality. Synthetic lethality describes a deadly synergistic effect on a cell due to the presence of two or more non-allelic mutations that are non-lethal on their own. However, the combined mutations within these specific genes will produce cell death. Synthetic lethality has become a promising strategy in cancer therapy to target mutations that are not present in normal cells. Genes can be targeted by small molecules or small RNAs, leading to loss of function, that becomes lethal when the complementary gene is mutated, i.e., in cancer cells only (Kaelin, 2005; Canaani, 2009; Scholl et al., 2009; Weidle et al., 2011). These complementarity targets may be part of (i) a uniquely redundant essential function; (ii) two subunits of an essential protein complex; (iii) the same essential pathways; or (iv) parallel pathways that are together essential for the studied phenotype (Kaelin, 2005). Complementary genes can be found experimentally from the investigation of hits obtained from high throughput screens of large compound or genome-wide RNAi collections (Dent et al., 2009; Suthers et al., 2009).

The synthetic lethality approach based on primary drug resistant cell lines has led to a multi-drug strategy to target secondary complementary lethal genes. Drug resistance might emerge from mutated cell lines after a long treatment period with a specific primary drug. Searching for secondary genes that are lethal for these resistant cell lines can be found by secondary screening of a compound collection against these resistant cell lines (Canaani, 2009; Dent et al., 2009). Attacking a complex disease such as cancer using drug combinations acting on multiple targets is likely to be more efficacious as the concept of synthetic lethality assumes a synergistic effect on essential targets or pathways. The synergistic action of drugs on multiple lethal genes is also less prone to drug resistance as it is more difficult for the cell to develop resistance simultaneously to targets that are also lethal complements to each other. Such therapeutic synergistic effects can be accompanied by synergistic side effects. However, large scale simulations of bacterial metabolism using multi-dose experiments relevant to diverse diseases, has provided evidence that synergistic drug combinations are generally more specific to particular cellular contexts than are single agent activities (Lehár et al., 2009; Neumann and Neumann-Staubitz, 2010). Moreover, synergistic combinations of two or more agents can overcome toxicity and other side effects associated with high doses of single drugs by countering biological compensation, allowing reduced dosage of each compound, or accessing context-specific multi-target mechanisms (Roth et al., 2004; Sharom et al., 2004; Kaelin, 2005; Keith et al., 2005; Hopkins, 2008).

Growing efforts on the experimental determination of synergy are being made in the field of polypharmacology to determine optimum drug combinations or dual drugs for pairs of synergistic targets that would increase the overall efficacy of disease treatment (Yildirim et al., 2007; Hopkins, 2008; Li et al., 2011; Csermely et al., 2013; Vitali et al., 2013). While synergistic toxicity may arise, drug combinations are expected to have less secondary effects due to amplified efficacy at lower doses for each drug. Synergy is evaluated experimentally by comparing with the action of the sum of the components using the Loewe additive model (Loewe, 1953), or the Bliss independence model (Bliss, 1956). A Drug Combination Index (Chou and Talalay, 1983) can be defined according to these two models depending whether the response of the drug is linearly related to the log of the dose or to the dose itself. Systematic investigation of relevant combinations of therapeutic targets can be done experimentally using cell-based screening on large sets of compounds (Canaani, 2009). However, this requires cell lines that have retained the essential phenotype of the disease as well as multi-dose preparations for all pairs of drug combinations or single-dose combinations of drug cocktails, as suggested by Tan et al. (2012). Computer approaches to predict highest scored combinations of targets or drugs that need to be validated experimentally are based either on topological graph analysis of biological networks (Vitali et al., 2013), as discussed in the previous section, or on flux balance analysis (FBA) of metabolic networks (Lehár et al., 2009), which are reviewed in more detail in the next section.

## Metabolic Synergies: Pathway Complementarities

Synergies are often found in organisms at the level of metabolism. Observed metabolic phenotypes in the organism’s metabolic network can be seen as emergent properties resulting from the tradeoff balance between competing objectives such as growth and energy consumption under Pareto’s optimality condition (Schuetz et al., 2012). Perturbations in the network will induce adjustments in order to achieve optimality under the new conditions. Synergies appear when the simultaneous perturbation of two nodes in a metabolic network induces a readjustment of flux distributions that corresponds to an augmented effect at a distant site in the network. Identifying such types of effects through FBA can be used either to design metabolic interventions increasing the production of a desired target metabolite in metabolic engineering or, in the opposite direction, to disrupt the metabolism of a pathogen or a cancer cell by multi-target and synthetic lethal drugs (Box 2). Synergies can also be observed at the systems level in the concerted operation of metabolic networks, like in the case of symbiotic and parasitic relationships (Christian et al., 2007) or in ecosystems (Klitgord and Segrè, 2011), where the metabolism of different species act in cooperation.

Box 2 **Finding synergies in metabolic engineering.**

In flux balance analysis (FBA), metabolic reactions are represented as a stoichiometric matrix **S**, where each row represents a compound and each column represents one reaction. The flux through the reactions in the network is represented by **v** and it is constrained between *v*^min^ and *v*^max^. FBA estimates the optimal flux solution that maximizes an objective function *v*_bio_ such as biomass, energy consumption, etc., expressed as a combination of fluxes, among the allowable space of flux distributions (Orth et al., 2010):

$$\begin{array}{llll} & \text{maximize}\;v_{\text{bio}}\; & & \;\\ & \text{subject}\;\text{to}\; & & \;\\ & \;\text{Sv }=\text{ 0}\; & & \;\\ & \;v_{i}^{\min}\leq v_{i}\leq v_{i}^{\max}\; & & \;\\ & \; & \end{array}$$

The solution to the previous constraint-based optimization can be found through the application of linear programing algorithms. Interestingly, in order to find synergies between nodes in the previous network that maximize (or minimize) the flux, *v_x_* associated with the desired activity, the optimization problem can be augmented into a mixed integer linear programing (MILP) problem where pairs, triplets, and so on of gene deletions are tested in order to find the best synergy that simultaneously maximize *v*_x_ and *v*_bio_, stated as follows:

$$\begin{array}{l} \underset{y_{i}}{\text{maximize }v_{x}}\\ \text{subject to}\\ \left[\begin{array}{l} \text{maximize }v_{\text{bio}}\\ \text{subject to}\\ \text{ Sv}=0\\ \;v_{i}^{\min}\leq v_{i}\leq v_{i}^{\max} \end{array}\right]\\ \sum_{i}\left(1-y_{i})=k,\;y_{i}=\{0,1\right\} \end{array}$$

where *K* is the number of allowable deletions. Depending upon the type of synergy (positive or negative), two types of applications are at least possible:

1. In synthetic lethality studies, *v_x_* is a negative flux that corresponds to the synergistic drug target, for instance in order to determine essential genes that impair growth in cancer cells (Folger et al., 2011).
2. In metabolic engineering, the goal is to introduce metabolic interventions such as gene knockouts that modify the fluxes so that the flux *v_x_* through the desired final product is maximized (Shen and Liao, 2013).

As discussed in the previous section, synergies are studied through metabolic networks in order to identify combinations of drug targets that have increased efficacy. For instance, compounds that inhibit enzymes in the pathway can target multiple enzymes in the pathway, since often they have conserved binding motifs interacting with similar metabolites acting as intermediates. This was the case for the shikimate pathway of *Helicobacter pylori*, where through a screening procedure compounds were found that could target both the shikimate dehydrogenase and shikimate kinase (Hsu et al., 2013). Studies of synthetic lethality can be performed *in silico* through FBA. In a reconstructed model of *Mycobacterium tuberculosis*, all pairs of double-deletion mutants (synthetic lethal pairs) were tested through FBA. Drugs associated with such synthetic lethal antimicrobial targets can represent drug synergy (Chavali et al., 2012). Similarly, the observation that many cancer cells adapt their metabolism has led to the identification of synthetic lethal drugs trough FBA of metabolic networks; these synergies were thereafter validated using available drug efficacy and gene expression measurements (Folger et al., 2011; McCarthy, 2011).

Synergies in metabolic networks are also a valuable tool for metabolic engineering. One of the implicit objectives of metabolic engineering is to employ synergies in order to achieve optimal production of a desired metabolite. Such synergies between metabolic pathways arise when the requirements of the pathways, such as energy, redox, or cofactor exchanges, are complementary (Shen and Liao, 2013). To that end, network analysis can be performed in order to determine simultaneous gene knockouts, knockdowns, and overexpression (knock-ins) in the host organism leading to overproduction of the target. For instance, a synergistic effect was observed on the production of *S*-adenosyl-l-methionine in *Pichia pastoris* by simultaneously using knocking in and knocking out techniques (He et al., 2006). In another example, a synergistic increase in plant oil levels was attained by simultaneously engineering genes involved in the biosynthesis of fatty acids (Vanhercke et al., 2013).

Such studies are based on the estimation of changes in the equilibrium of fluxes through FBA when multiple genes encoding enzymes are deleted in the network (Ranganathan et al., 2010). In addition, synergies in metabolic engineering can also be obtained through synergistic fine control of gene expression of the enzymes in the pathway in order to allow the reduction of bottlenecks and of growth inhibition from accumulation of intermediates (Flowers et al., 2013). Developing regulatory parts such as promoters, ribosome binding sites (RBS), and riboswitches have been shown to be useful for achieving such synergies (Arpino et al., 2013). Furthermore, another type of synergy that is naturally found in metabolic pathways are the ones induced by enzyme co-localization when reactions are brought into close proximity through a multifunction enzyme that couples sequential conversion steps in the pathway, allowing by this means substrate channeling. Benefits are multiple; namely, enzyme co-localization reduces intermediate’s diffusion distance, can keep local concentrations high, and can reduce cross-talk between competing pathways. Many examples exist where synergistic improvements in comparison with individual enzyme activities were obtained through co-localization (Conrado et al., 2008). Interestingly, this strategy has been mimicked by engineers in the design of synthetic protein scaffolds that physically link enzymes in a way that allowed the effective concentration of each component of the desired pathway to be increased (Dueber et al., 2009).

## Synergistic Design for Synthetic Biology Innovation

**Figure 3:**
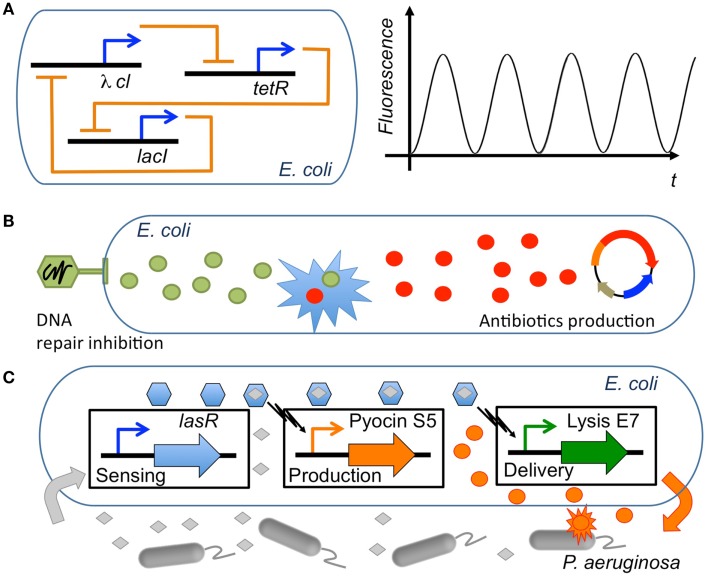
**Synergies in synthetic biology applications**. Several types of synergies used in synthetic biology circuits engineered in *E. coli*. **(A)** Constitutive synergy: the repressilator (Elowitz and Leibler, 2000) represents a type of genetic circuit with constitutive synergy, where a cycle of three repressor proteins causes a synergy leading the network to oscillate (adapted from Chandran et al., 2008). **(B)** Induced synergy: an engineered bacteriophage that overexpresses *lexA3* to suppress the SOS DNA repair (Lu and Collins, 2009) can induce the synergistic effect of lowering tolerance to antibiotics. Thus, the antimicrobial effect of an engineered genetic circuit producing antibiotics (Planson et al., 2011) can be significantly increased. **(C)** Modular synergy: a combination of three modules that sense the presence of *P. aeruginosa*, produce the antibiotic pyocin S5 and deliver the antimicrobial by triggering lysis of the cell, respectively, acts synergistically to kill the pathogen when its presence is detected (Saeidi et al., 2011).

Synergy is a concept that permeates much of synthetic biology. Although in a rather non-systematic way, synthetic biology has often taken advantage of existing synergies, either natural or those found in experimental screening, in order to create new behavior from the combination of biological elements (Agapakis and Silver, 2009; Khalil and Collins, 2010). Synergies in synthetic biology applications can be grouped into three main types (Table 2; Figure 3): (i) constitutive synergies, which are found when the emergent behavior of the device comes from synergies among its internal elements; (ii) induced synergies, which occur if the output activity of two or more synthetic biology devices induce a synergistic effect in a host or external organism; (iii) modular synergies, which appear when the independent behavior of multiple modules gives birth to a synergistic outcome once they are interconnected.

**Table 2:** **Examples of synthetic biology synergistic applications**.

Type of synergy	Elements showing synergistic effects	Main analysis and design methodologies	Synthetic biology applications
Constitutive	Circuit’s genetic parts	Genetic circuit design	Toggle switch (Gardner et al., 2000)
			Repressilator (Elowitz and Leibler, 2000)
Induced	Organism’s biological elements (drugs, mutations, pathways, etc.)	Systems biology	Combinatorial antibiotic treatment (Lu and Collins, 2009; Weber and Fussenegger, 2009; Kohanski et al., 2010)
		Network analysis	Synergic treatment of metabolic disorders (Ye et al., 2013)
		Metabolic network analysis	Bistability in bacterial populations (Tan et al., 2009)
Modular	Synthetic modules	Biosensor design	Auxin biosensor from a mammalian and a plant circuit (Wend et al., 2013)
		Interface design	Sensor and delivery for killing pathogens (Saeidi et al., 2011)
		Systems and control theory	Analog (Daniel et al., 2013) and multicellular computation (Goñi-Moreno et al., 2013)

Constitutive synergy is established between parts of a synthetic biology module in order to provide its desired function. They are obtained by the skillful application of genetic circuit design principles. Such cooperative interactions among genetic parts enabled many of the early synthetic biology devices. Among others, toggle switches (Gardner et al., 2000) and oscillator gene circuits such as the repressilator (Elowitz and Leibler, 2000) were early examples of gene cooperativity giving birth to a well-defined emergent behavior. The resulting cooperative behavior between genes is most often linked to feedback relationships (To and Maheshri, 2010; Jayanthi et al., 2013), i.e., genes that are combined through repression/activation interconnections in a way that allows generation of the desired synthetic behavior (Rollie et al., 2012). In order to establish a synergistic effect between the genes, properties for each part need to be carefully selected. For instance, it has been shown that to obtain oscillations, it is necessary to impose a time-scale design condition with a repressor that is sufficiently slow in comparison with the activator (Jayanthi and Del Vecchio, 2012). Once a constitutive synergy has been appropriately designed and tuned in the circuit, the resulting functional device can potentially be employed in multiple synthetic biology applications.

A second level of synergy used in synthetic biology is induced synergy, i.e., synergy that is created between the outputs of synthetic biology devices. As discussed in previous sections, many useful synergistic events in biological systems have been identified through systems biology and network analysis. Synthetic biology modules are here used in cooperation in order to trigger external synergistic events (either in the host organism or in a external pathogen) that might be useful for biological applications. For instance, to this category belong applications that use drug synergies through the delivery of multiple drugs by synthetic biology devices. Essential genes appear here as a basic tool used in order to identify synergistic targets, for example with antimicrobial activity (Juhas et al., 2012). One example is the use of engineered bacteriophages to attack gene networks that are not directly targeted by antibiotics (Lu and Collins, 2009; Lu et al., 2009). By modifying the oxidative stress response or the inhibition of DNA damage repair systems in *Escherichia coli*, the antimicrobial effect of several antibiotics such as quinolone, gentamicin, and ampicillin was enhanced. Furthermore, control of biofilm formation and inhibition of mechanisms involved in antibiotic resistance were also enhanced by the engineered bacteriophages (Weber and Fussenegger, 2009; Kohanski et al., 2010). In yet another example, a first molecule (guanabenz) was used in order to activate a synthetic signal cascade that stimulated the secretion of a fusion protein that regulates the metabolism based on the synergistic action of a peptide (GLP-1) and the hormone leptin (Ye et al., 2013). The authors showed that it was by this means possible to regulate the metabolic syndrome states linked to several diseases (obesity, hypertension, hyperglycemia, etc.). Besides these examples of induced synergy between the outputs of synthetic biology circuits, other induced synergies might appear between the inserted circuit and the host organism because of variations in global host resources (Cardinale et al., 2013) or cell growth (Scott et al., 2010), leading to different circuit performances. Interestingly, such synergistic effects have been used to generate bistability in bacterial populations through growth-rate dependent gene expression (Tan et al., 2009; Klumpp, 2011), opening the possibility to another type of synthetic biology applications based on the induced synergy between circuits and the host organism.

In the third group of synthetic biology synergies belong those that emerge at the modular level, i.e., emergent behavior elicited by the concerted operation of multiple synthetic biology devices. If the two previously described groups of synergy were established either internally (constitutive) or externally (induced) with respect to the inserted synthetic biology modules, modular synergy is a synergy that is specifically established between the modules. Here, each synthetic biology module operates in a rather independent or orthogonal way from the others except at the input/output interface where the actual synergy is formed. The resulting circuit interferences or cross-talk can be characterized through properties such as retroactivity, a property analog to output impedance in electronic circuits (Del Vecchio et al., 2008; Jiang et al., 2011), and fan-out (Kim and Sauro, 2010). Theoretical studies have shown that these properties can be finely tuned through an appropriate insulation and time-scale separation (Jayanthi and Del Vecchio, 2011). For instance, insulation mechanisms for module interfaces have been proposed based on phosphorylation-dephosphorylation cycles (Del Vecchio et al., 2008), the appropriate selection of DNA target sites for transcription factors (Jayanthi and Del Vecchio, 2012) or through the signaling metabolite acetyl phosphate in order to generate oscillations (Fung et al., 2005). In general, introducing delays at the circuit’s interface through transcription and translation control (riboregulators, RBS, etc.) (Purnick and Weiss, 2009), regulatory regions, degradation, inducible promoter regulation (Jayanthi and Del Vecchio, 2012) or enzymatic processes (Cookson et al., 2011) constitute flexible methods to tune synthetic biology circuits. These genetic elements work very often in a synergistic way (Perez-Pinera et al., 2013). Therefore, models that precisely estimate synergistic activities are needed in the synthetic biology design toolbox. Some examples are operon calculators (Salis et al., 2009) as well as others ways of controlling gene expression from genetic elements (Meng et al., 2013; Mutalik et al., 2013). Synthetic biology applications that have used modular synergies include for instance an *E. coli* strain that was engineered to sense and kill *Pseudomona aeruginosa*. A first module consisted of a sensing device that detected the presence of *P. aeruginosa* through its quorum sensing signal. A second module was responsible for the production of pyocin, an antibiotic and of E7 lysis protein causing the chassis to lyse and resulting in the release of pyocin (Saeidi et al., 2011). In this application, the concerted operation of both modules resulted in the killing activity. In another example, a synergistic mammalian and plant synthetic biology approach was used to develop a ratiometric luminescent biosensor with wide applications including study of auxin metabolism, transport, and signaling (Wend et al., 2013). More advanced applications of modular synergy are circuits performing complex computations, such as synthetic analog gene circuits (Daniel et al., 2013) and multicellular computation (Goñi-Moreno et al., 2013).

## Perspectives

Synergies have been driving innovation both in nature and in synthetic designs. They have served to implement innovative solutions in fields as diverse as drug design, where drug synergies provided new ways to overcome urgent problems such as resistance, and in metabolic networks, where synergies have found their way in pathway complementarity toward amplified targeted effects. By pushing forward the concept into synthetic biology, we would argue that new promising applications may emerge in the near future.

To date, synergy has already been successfully used in synthetic biology to induce non-natural behavior in synthetic constructs, to empower the outcome of biological circuits, or to enable the concerted operation of modules into an emergent function. The next frontier will be in the development of a systematic characterization of synergistic effects in synthetic biology devices in a similar manner as synergies are characterized through modeling and screening in other disciplines such as drug design and metabolism. Beyond the concept of orthogonality in modular design, synergistic cross-talk between modules arises here as a valuable feature that should be appropriately characterized in the specifications found in biological circuit catalogs. The adoption of such proposed practice of synergistic synthetic biology, thus, will require a design paradigm shift toward circuit topologies that will deliberately display the ability of emergent behavior once operating in concert.

## Conflict of Interest Statement

The authors declare that the research was conducted in the absence of any commercial or financial relationships that could be construed as a potential conflict of interest.

## References

[B1] Agapakis C. M. Silver P. A. (2009). Synthetic biology: exploring and exploiting genetic modularity through the design of novel biological networks. Mol. Biosyst. 5, 704–713 10.1039/b901484e 19562109

[B2] Alon U. (2003). Biological networks: the tinkerer as an engineer. Science 301, 1866–1867 10.1126/science.1089072 14512615

[B3] Anastassiou D. (2007). Computational analysis of the synergy among multiple interacting genes. Mol. Syst. Biol. 3 10.1038/msb4100124 17299419 PMC1828751

[B4] Arpino J. A. J. Hancock E. J. Anderson J. Barahona M. Stan G.-B. V. Papachristodoulou A. (2013). Tuning the dials of synthetic biology. Microbiology 159(Pt 7), 1236–1253 10.1099/mic.0.067975-0 23704788 PMC3749727

[B5] Atkinson M. Savageau M. Myers J. Ninfa A. (2003). Development of genetic circuitry exhibiting toggle switch or oscillatory behavior in *Escherichia coli*. Cell 13, 597–607 10.1016/S0092-8674(03)00346-5 12787501

[B6] Bell-Pedersen D. Cassone V. M. Earnest D. J. Golden S. S. Hardin P. E. Thomas T. L. (2005). Circadian rhythms from multiple oscillators: lessons from diverse organisms. Nat. Rev. Genet. 6, 544–556 10.1038/nrg1633 15951747 PMC2735866

[B7] Benner S. A. Sismour A. M. (2005). Synthetic biology. Nat. Rev. Genet. 6, 533–543 10.1038/nrg1637 15995697 PMC7097405

[B8] Bliss C. I. (1956). The calculation of microbial assays. Bacteriol. Rev. 20, 243–258 13403845 10.1128/br.20.4.243-258.1956 PMC180866

[B9] Canaani D. (2009). Methodological approaches in application of synthetic lethality screening towards anticancer therapy. Br. J. Cancer 100, 1213–1218 10.1038/sj.bjc.6605000 19319136 PMC2676542

[B10] Cardinale S. Joachimiak M. P. Arkin A. P. (2013). Effects of genetic variation on the *E. coli* host-circuit interface. Cell Rep. 4, 231–237 10.1016/j.celrep.2013.06.023 23871664

[B11] Challis G. L. Hopwood D. A. (2003). Synergy and contingency as driving forces for the evolution of multiple secondary metabolite production by *Streptomyces* species. Proc. Natl. Acad. Sci. U.S.A. 100(Suppl. 2), 14555–14561 10.1073/pnas.1934677100 12970466 PMC304118

[B12] Chandran D. Copeland W. B. Sleight S. C. Sauro H. M. (2008). Mathematical modeling and synthetic biology. Drug Discov. Today Dis. Models 5, 299–309 10.1016/j.ddmod.2009.07.002 PMC5102263 27840651

[B13] Chavali A. K. D’Auria K. M. Hewlett E. L. Pearson R. D. Papin J. A. (2012). A metabolic network approach for the identification and prioritization of antimicrobial drug targets. Trends Microbiol. 20, 113–123 10.1016/j.tim.2011.12.004 22300758 PMC3299924

[B14] Chou T. C. Talalay P. (1983). Analysis of combined drug effects: a new look at a very old problem. Trends Pharmacol. Sci. 4, 450–454 10.1016/0165-6147(83)90490-X

[B15] Christian N. Handorf T. Ebenhh O. (2007). Metabolic synergy: increasing biosynthetic capabilities by network cooperation. Genome Inform. 18, 320–329 10.1142/9781860949920_0031 18546499

[B16] Chua H. E. Zhao Q. Bhowmick S. S. Dewey C. F. Tucker-Kellog L. Yu H. (2011). “Pani: a novel algorithm for fast discovery of putative target nodes in signaling networks,” in Proceedings of the 2nd ACM Conference on Bioinformatics Computational Biology and Biomedicine (New York: ACM), 284–288 10.1145/2147805.2147836

[B17] Cokol M. Chua H. N. N. Tasan M. Mutlu B. Weinstein Z. B. Suzuki Y. (2011). Systematic exploration of synergistic drug pairs. Mol. Syst. Biol. 7 10.1038/msb.2011.71 22068327 PMC3261710

[B18] Conrado R. J. Varner J. D. DeLisa M. P. (2008). Engineering the spatial organization of metabolic enzymes: mimicking nature’s synergy. Curr. Opin. Biotechnol. 19, 492–499 10.1016/j.copbio.2008.07.006 18725290

[B19] Cookson N. A. Mather W. H. Danino T. Mondragon-Palomino O. Williams R. J. Tsimring L. S. (2011). Queueing up for enzymatic processing: correlated signaling through coupled degradation. Mol. Syst. Biol. 7 10.1038/msb.2011.94 22186735 PMC3737734

[B20] Corning P. A. (2013). Systems theory and the role of synergy in the evolution of living systems. Syst. Res. 10.1002/sres.2191

[B21] Csermely P. Korcsmáros T. Kiss H. J. M. London G. Nussinov R. (2013). Structure and dynamics of molecular networks: a novel paradigm of drug discovery. A comprehensive review. Pharmacol. Ther. 138, 333–408 10.1016/j.pharmthera.2013.01.016 23384594 PMC3647006

[B22] Daniel R. Rubens J. R. Sarpeshkar R. Lu T. K. (2013). Synthetic analog computation in living cells. Nature 497, 619–623 10.1038/nature12148 23676681

[B23] Del Vecchio D. Ninfa A. J. Sontag E. D. (2008). Modular cell biology: retroactivity and insulation. Mol. Syst. Biol. 4, 161 10.1038/msb4100204 18277378 PMC2267736

[B24] Dent P. Curiel D. T. Fisher P. B. Grant S. (2009). Synergistic combinations of signaling pathway inhibitors: mechanisms for improved cancer therapy. Drug Resist. Updates 12, 65–73 10.1016/j.drup.2009.03.001 19395305 PMC2696566

[B25] Dueber J. E. Wu G. C. Malmirchegini G. R. Moon T. S. Petzold C. J. Ullal A. V. (2009). Synthetic protein scaffolds provide modular control over metabolic flux. Nat. Biotechol. 27, 753–759 10.1038/nbt.1557 19648908

[B26] Elowitz M. B. Leibler S. (2000). A synthetic oscillatory network of transcriptional regulators. Nature 403, 335–338 10.1038/35002125 10659856

[B27] Endy D. (2005). Foundations for engineering biology. Nature 438, 449–453 10.1038/nature04342 16306983

[B28] Feynman R. P. (1988). QED: The Strange Theory of Light and Matter. Princeton: Princeton University Press, 176

[B29] Flowers D. Thompson R. A. Birdwell D. Wang T. Trinh C. T. (2013). SMET: systematic multiple enzyme targeting a method to rationally design optimal strains for target chemical overproduction. Biotechnol. J. 8, 605–618 10.1002/biot.201200233 23613435

[B30] Folger O. Jerby L. Frezza C. Gottlieb E. Ruppin E. Shlomi T. (2011). Predicting selective drug targets in cancer through metabolic networks. Mol. Syst. Biol. 7 10.1038/msb.2011.35 PMC3159974 21694718

[B31] Fung E. Wong W. W. Suen J. K. Bulter T. Lee S. Liao J. C. (2005). A synthetic gene-metabolic oscillator. Nature 435, 118–122 10.1038/nature03508 15875027

[B32] Gardner T. S. Cantor C. R. Collins J. J. (2000). Construction of a genetic toggle switch in *Escherichia coli*. Nature 403, 339–342 10.1038/35002131 10659857

[B33] Goñi-Moreno A. Amos M. de la Cruz F. (2013). Multicellular computing using conjugation for wiring. PLoS ONE 8:e65986 10.1371/journal.pone.0065986 23840385 PMC3688716

[B34] Hartwell L. H. Hopfield J. J. Leibler S. Murray A. W. (1999). From molecular to modular cell biology. Nature 402, 47–52 10.1038/46972 10591225

[B35] He J. Deng J. Zheng Y. Gu J. (2006). A synergistic effect on the production of S-adenosyl-L-methionine in *Pichia pastoris* by knocking in of S-adenosyl-L-methionine synthase and knocking out of cystathionine-beta synthase. J. Biotechnol. 126, 519–527 10.1016/j.jbiotec.2006.05.009 16828189

[B36] Hegreness M. Shoresh N. Damian D. Hartl D. Kishony R. (2008). Accelerated evolution of resistance in multidrug environments. Proc. Natl. Acad. Sci. U.S.A. 105, 13977–13981 10.1073/pnas.0805965105 18779569 PMC2544564

[B37] Hess B. (2000). Periodic patterns in biology. Naturwissenschaften 87, 199–211 10.1007/s001140050704 10883434

[B38] Hopkins A. L. (2008). Network pharmacology: the next paradigm in drug discovery. Nat. Chem. Biol. 4, 682–690 10.1038/nchembio.118 18936753

[B39] Hsu K. Cheng W. Chen Y. (2013). Pathway-based screening strategy for multitarget inhibitors of diverse proteins in metabolic pathways. PLoS Comput. Biol. 9:e1003127 10.1371/journal.pcbi.1003127 23861662 PMC3701698

[B40] Hwang W. Kim T. Ramanathan M. Zhang A. (2008). “Bridging centrality: graph mining from element level to group level,” in Proceedings of the 14th ACM SIKDD International Conference on Knowledge Discovery and Data Mining (New York: ACM), 336–344 10.1145/1401890.1401934

[B41] Ihekwaba A. E. Broomhead D. S. Grimley R. Benson N. White M. R. Kell D. B. (2005). Synergistic control of oscillations in the NF-kappaB signalling pathway. Syst. Biol. 152, 153–160 10.1049/ip-syb:20050050 16986278

[B42] Jayanthi S. Del Vecchio D. (2011). Retroactivity attenuation in bio-molecular systems based on timescale separation. IEEE Trans. Automat. Contr. 56, 748–761 10.1109/TAC.2010.2069631

[B43] Jayanthi S. Del Vecchio D. (2012). Tuning genetic clocks employing DNA binding sites. PLoS ONE 7:e41019 10.1371/journal.pone.0041019 22859962 PMC3409220

[B44] Jayanthi S. Nilgiriwala K. S. Del Vecchio D. (2013). Retroactivity controls the temporal dynamics of gene transcription. ACS Synth. Biol. 2, 431–441 10.1021/sb300098w 23654274

[B45] Jiang P. Ventura A. C. Sontag E. D. Merajver S. D. Ninfa A. J. Del Vecchio D. (2011). Load-induced modulation of signal transduction networks. Sci. Signal. 4, ra67 10.1126/scisignal.2002152 21990429 PMC8760836

[B46] Juhas M. Eberl L. Church G. M. (2012). Essential genes as antimicrobial targets and cornerstones of synthetic biology. Trends Biotechnol. 30, 601–607 10.1016/j.tibtech.2012.08.002 22951051

[B47] Kadanoff L. P. (2013). Kenneth Geddes Wilson (1936–2013). Nature 500, 30 10.1038/500030a 23903743

[B48] Kaelin W. G. (2005). The concept of synthetic lethality in the context of anticancer therapy. Nat. Rev. Cancer 5, 689–698 10.1038/nrc1691 16110319

[B49] Keith C. T. Borisy A. A. Stockwell B. R. (2005). Multicomponent therapeutics for networked systems. Nat. Rev. Drug Discov. 4, 71–78 10.1038/nrd1609 15688074

[B50] Khalil A. S. Collins J. J. (2010). Synthetic biology: applications come of age. Nat. Rev. Genet. 11, 367–379 10.1038/nrg2775 20395970 PMC2896386

[B51] Kim K. Sauro H. (2010). Fan-out in gene regulatory networks. J. Biol. Eng. 4, 16 10.1186/1754-1611-4-16 21167053 PMC3024275

[B52] Kitney R. Freemont P. (2012). Synthetic biology – the state of play. FEBS Lett. 586, 2029–2036 10.1016/j.febslet.2012.06.002 22704968

[B53] Klitgord N. Segrè D. (2011). Ecosystems biology of microbial metabolism. Curr. Opin. Biotechnol. 22, 541–546 10.1016/j.copbio.2011.04.018 21592777

[B54] Klumpp S. (2011). Growth-rate dependence reveals design principles of plasmid copy number control. PLoS ONE 6:e20403 10.1371/journal.pone.0020403 21647376 PMC3103578

[B55] Kohanski M. A. Dwyer D. J. Collins J. J. (2010). How antibiotics kill bacteria: from targets to networks. Nat. Rev. Microbiol. 8, 423–435 10.1038/nrmicro2333 20440275 PMC2896384

[B56] Kotlyar M. Fortney K. Jurisica I. (2012). Network-based characterization of drug-regulated genes, drug targets, and toxicity. Methods 57, 499–507 10.1016/j.ymeth.2012.06.003 22749929

[B57] Lanza A. M. Crook N. C. Alper H. S. (2012). Innovation at the intersection of synthetic and systems biology. Curr. Opin. Biotechnol. 23, 712–717 10.1016/j.copbio.2011.12.026 22265125

[B58] Lehár J. Krueger A. S. Avery W. Heilbut A. M. Johansen L. M. Price E. R. (2009). Synergistic drug combinations tend to improve therapeutically relevant selectivity. Nat. Biotechnol. 27, 659–666 10.1038/nbt.1549 19581876 PMC2708317

[B59] Li S. Zhang B. Zhang N. (2011). Network target for screening synergistic drug combinations with application to traditional Chinese medicine. BMC Syst. Biol. 5:S10 10.1186/1752-0509-5-S1-S10 21689469 PMC3121110

[B60] Loewe S. (1953). The problem of synergism and antagonism of combined drugs. Arzneimittelforschung 3, 285–290 13081480

[B61] Lu T. K. Collins J. J. (2009). Engineered bacteriophage targeting gene networks as adjuvants for antibiotic therapy. Proc. Natl. Acad. Sci. U.S.A. 106, 4629–4634 10.1073/pnas.0800442106 19255432 PMC2649960

[B62] Lu T. K. Khalil A. S. Collins J. J. (2009). Next-generation synthetic gene networks. Nat. Biotechnol. 27, 1139–1150 10.1038/nbt.1591 20010597 PMC2796205

[B63] Lucks J. B. Qi L. Whitaker W. R. Arkin A. P. (2008). Toward scalable parts families for predictable design of biological circuits. Curr. Opin. Microbiol. 11, 567–573 10.1016/j.mib.2008.10.002 18983935

[B64] Mani R. St Onge R. P. Hartman J. L. Giaver G. (2008). Defining genetic interaction. Proc. Natl. Acad. Sci. U.S.A. 105, 2461–3466 10.1073/pnas.0712255105 PMC2265146 18305163

[B65] McCarthy N. (2011). Systems biology: lethal weaknesses. Nat. Rev. Cancer 11, 538–539 10.1038/nrc3109 21734723

[B66] Meng H. Wang J. Xiong Z. Xu F. Zhao G. Wang Y. (2013). Quantitative design of regulatory elements based on high-precision strength prediction using artificial neural network. PLoS ONE 8:e60288 10.1371/journal.pone.0060288 23560087 PMC3613377

[B67] Mutalik V. K. Guimaraes J. C. Cambray G. Lam C. Christoffersen M. J. Mai Q.-A. (2013). Precise and reliable gene expression via standard transcription and translation initiation elements. Nat. Methods 10, 354–360 10.1038/nmeth.2404 23474465

[B68] Neumann H. Neumann-Staubitz P. (2010). Synthetic biology approaches in drug discovery and pharmaceutical biotechnology. Appl. Microbiol. Biotechnol. 87, 75–86 10.1007/s00253-010-2578-3 20396881 PMC2872025

[B69] Nordwald E. M. Garst A. Gill R. T. Kaar J. L. (2013). Accelerated protein engineering for chemical biotechnology via homologous recombination. Curr. Opin. Biotechnol. 10.1016/j.copbio.2013.03.003 23540421

[B70] Orth J. D. Thiele I. Palsson B. O. (2010). What is flux balance analysis? Nat. Biotechnol. 28, 245–248 10.1038/nbt.1614 20212490 PMC3108565

[B71] Perez-Pinera P. Ousterout D. G. Brunger J. M. Farin A. M. Glass K. A. Guilak F. (2013). Synergistic and tunable human gene activation by combinations of synthetic transcription factors. Nat. Methods 10, 239–242 10.1038/nmeth.2361 23377379 PMC3719416

[B72] Planson A.-G. Carbonell P. Grigoras I. Faulon J.-L. (2011). Engineering antibiotic production and overcoming bacterial resistance. Biotechnol. J. 6, 812–825 10.1002/biot.201100085 21661120

[B73] Purnick P. E. M. Weiss R. (2009). The second wave of synthetic biology: from modules to systems. Nat. Rev. Mol. Cell Biol. 10, 410–422 10.1038/nrm2698 19461664

[B74] Ranganathan S. Suthers P. F. Maranas C. D. (2010). OptForce: an optimization procedure for identifying all genetic manipulations leading to targeted overproductions. PLoS Comput. Biol. 6:e1000744 10.1371/journal.pcbi.1000744 20419153 PMC2855329

[B75] Rollie S. Mangold M. Sundmacher K. (2012). Designing biological systems: systems engineering meets synthetic biology. Chem. Eng. Sci. 69, 1–29 10.1016/j.ces.2011.10.068

[B76] Roth B. Sheffler D. Kroeze W. K. (2004). Magic shotguns versus magic bullets: selectively non-selective drugs for mood disorders and schizophrenia. Nat. Rev. Drug Discov. 3, 353–359 10.1038/nrd1346 15060530

[B77] Saeidi N. Wong C. K. Lo T.-M. Nguyen H. X. Ling H. Leong S. S. (2011). Engineering microbes to sense and eradicate *Pseudomonas aeruginosa*, a human pathogen. Mol. Syst. Biol. 7, 521 10.1038/msb.2011.55 21847113 PMC3202794

[B78] Salis H. M. Mirsky E. A. Voigt C. A. (2009). Automated design of synthetic ribosome binding sites to control protein expression. Nat. Biotechnol. 27, 946–950 10.1038/nbt.1568 19801975 PMC2782888

[B79] Scholl C. Fröhling S. Dunn I. F. Schinzel A. C. Barbie D. A. Kim S. Y. (2009). Synthetic lethal interaction between oncogenic KRAS dependency and STK33 suppression in human cancer cells. Cell 137, 821–834 10.1016/j.cell.2009.03.017 19490892

[B80] Schuetz R. Zamboni N. Zampieri M. Heinemann M. Sauer U. (2012). Multidimensional optimality of microbial metabolism. Science 336, 601–604 10.1126/science.1216882 22556256

[B81] Scott M. Gunderson C. W. Mateescu E. M. Zhang Z. Hwa T. (2010). Interdependence of cell growth and gene expression: origins and consequences. Science 330, 1099–1102 10.1126/science.1192588 21097934

[B82] Sharom J. R. Bellows D. S. Tyers M. (2004). From large networks to small molecules. Curr. Opin. Chem. Biol. 8, 81–90 10.1016/j.cbpa.2003.12.007 15036161

[B83] Shen C. R. Liao J. C. (2013). Synergy as design principle for metabolic engineering of 1-propanol production in *Escherichia coli*. Metab. Eng. 17, 12–22 10.1016/j.ymben.2013.01.008 23376654

[B84] Stricker J. Cookson S. Bennett M. R. Mather W. H. Tsimring L. S. Hasty J. (2008). A fast, robust and tunable synthetic gene oscillator. Nature 456, 516–519 10.1038/nature07389 18971928 PMC6791529

[B85] Suthers P. F. Zomorrodi A. Maranas C. D. (2009). Genome-scale gene/reaction essentiality and synthetic lethality analysis. Mol. Syst. Biol. 5, 301 10.1038/msb.2009.56 19690570 PMC2736653

[B86] Tan C. Marguet P. You L. (2009). Emergent bistability by a growth-modulating positive feedback circuit. Nat. Chem. Biol. 5, 842–848 10.1038/nchembio.218 19801994 PMC2908482

[B87] Tan X. Hu L. Luquette L. J. Gao G. Liu Y. Qu H. (2012). Systematic identification of synergistic drug pairs targeting HIV. Nat. Biotechnol. 30, 1125–1130 10.1038/nbt.2391 23064238 PMC3494743

[B88] Tigges M. Marquez-Lago T. Stelling J. Fussenegger M. (2009). A tunable synthetic mammalian oscillator. Nature 457, 309–312 10.1038/nature07616 19148099

[B89] To T.-L. Maheshri N. (2010). Noise can induce bimodality in positive transcriptional feedback loops without bistability. Science 327, 1142–1145 10.1126/science.1178962 20185727

[B90] Topkis D. M. (1998). Supermodularity and Complementarity. Princeton: Princeton University Press

[B91] Vanhercke T. Tahchy A. El Shrestha P. Zhou X.-R. Singh S. P. Petrie J. R. (2013). Synergistic effect of WRI1 and DGAT1 coexpression on triacylglycerol biosynthesis in plants. FEBS Lett. 587, 364–369 10.1016/j.febslet.2012.12.018 23313251

[B92] Vitali F. Mulas F. Marini P. Bellazzi R. (2013). Network-based target ranking for polypharmacological therapies. J. Biomed. Inform. 46, 876–881 10.1016/j.jbi.2013.06.015 23850841

[B93] Wang B. Buck M. (2012). Customizing cell signaling using engineered genetic logic circuits. Trends Microbiol. 20, 376–384 10.1016/j.tim.2012.05.001 22682075

[B94] Weber W. Fussenegger M. (2009). The impact of synthetic biology on drug discovery. Drug Discov. Today 14, 956–963 10.1016/j.drudis.2009.06.010 19580884 PMC7108258

[B95] Weidle U. H. Maisel D. Eick D. (2011). Synthetic lethality-based targets for discovery of new cancer therapeutics. Cancer Genomics Proteomics 8, 159–171 21737609

[B96] Wend S. Bosco C. D. Kampf M. M. Ren F. Palme K. Weber W. (2013). A quantitative ratiometric sensor for time-resolved analysis of auxin dynamics. Sci. Rep. 3 10.1038/srep02052 23787479 PMC3689175

[B97] Wintermute E. H. Silver P. A. (2010). Emergent cooperation in microbial metabolism. Mol. Syst. Biol. 6 10.1038/msb.2010.66 20823845 PMC2964121

[B98] Ye H. Hamri G. C. Zwicky K. Christen M. Folcher M. Fussenegger M. (2013). Pharmaceutically controlled designer circuit for the treatment of the metabolic syndrome. Proc. Natl. Acad. Sci. U.S.A. 110, 141–146 10.1073/pnas.1216801110 23248313 PMC3538262

[B99] Yeomans J. M. (1992). Statistical Mechanics of Phase Transitions (Oxford Science Publications). Oxford: Clarendon Press, 164

[B100] Yildirim M. A. Goh K. Cusick M. E. Barabási A. Vidal M. (2007). Drug – target network. Nat. Biotechnol. 25, 1119–1126 10.1038/nbt1338 17921997

